# Thoracoscopic Removal of a Fish Bone Retained in the Lung for Five Years without the Need for Lung Resection: A Case Report

**DOI:** 10.70352/scrj.cr.25-0467

**Published:** 2025-12-02

**Authors:** Takaaki Nakatsukasa, Ryotaro Kamohara, Yasuhiro Tanaka, Takuto Miyamura, Yasuhiro Umeyama, Yasushi Obase, Hiroko Hayashi, Takuro Miyazaki, Keitaro Matsumoto, Akihiro Nakamura

**Affiliations:** 1 Department of Thoracic Surgery, Sasebo City General Hospital, Sasebo, Nagasaki, Japan; 2 Department of Pulmonology, Sasebo City General Hospital, Sasebo, Nagasaki, Japan; 3 Department of Diagnostic Pathology, Sasebo City General Hospital, Sasebo, Nagasaki, Japan; 4 Department of General Thoracic Surgery, Nagasaki University Hospital, Nagasaki, Nagasaki, Japan

**Keywords:** fish bone, foreign body, video-assisted thoracoscopic surgery (VATS), lung migration

## Abstract

**INTRODUCTION:**

The accidental ingestion of fish bones is a common clinical occurrence, especially in regions with high fish consumption. While most foreign bodies pass uneventfully or are removed endoscopically, sharp objects such as fish bones may perforate the gastrointestinal tract and migrate to adjacent organs, occasionally leading to severe complications. Migration into the lung parenchyma is extremely rare.

**CASE PRESENTATION:**

An 80-year-old woman presented with a 5-year history of intermittent hemoptysis following an episode initially diagnosed as bacterial pneumonia. CT revealed a linear hyperdense structure suggestive of a retained fish bone that had penetrated the esophageal wall and migrated into the peripheral left lower lobe. The foreign body had gradually shifted peripherally over time. Video-assisted thoracoscopic surgery (VATS) was performed, and the fish bone, measuring 4 cm, was successfully extracted through a small incision in the lung parenchyma without the need for lobectomy. The postoperative course was uneventful, and follow-up imaging showed resolution of the surrounding inflammation.

**CONCLUSIONS:**

This case highlights an extremely rare instance of a fish bone that remained in the lung for 5 years after esophageal perforation. It underscores the importance of considering retained foreign bodies in the differential diagnosis of unexplained pulmonary symptoms and imaging findings. Furthermore, it demonstrates that even long-standing foreign bodies may be managed effectively with a minimally invasive, lung-preserving approach when preoperative evaluation is carefully conducted.

## Abbreviations

CRP
: C-reactive protein
LLL
: left lower lung
VATS
: video-assisted thoracoscopic surgery

## INTRODUCTION

Foreign body ingestion is a common occurrence in daily clinical practice, with the majority of cases resolving spontaneously or being managed successfully with endoscopic removal.^1)^ However, in rare instances, the ingested object may perforate the gastrointestinal tract, leading to severe complications.^2)^ Among such cases, fish bones are frequently implicated due to their sharp structure and the high prevalence of fish consumption in certain cultures.^3)^

There have been reports of fish bones perforating the esophagus and migrating into the mediastinum or thoracic cavity, but cases in which the foreign body penetrates into the lung parenchyma are exceedingly rare. In some of these instances, the foreign body may remain in the lung for an extended period, causing chronic inflammation, recurrent pneumonia, or hemoptysis.

Here, we present an extremely rare case of a fish bone that perforated the gastrointestinal tract and migrated into the lung parenchyma, where it remained for 5 years. Remarkably, the foreign body was successfully removed thoracoscopically without the need for pulmonary resection.

## CASE PRESENTATION

An 80-year-old woman presented to a local clinic with upper abdominal pain 5 years earlier and was diagnosed with bacterial pneumonia based on CT. Laboratory data revealed a white blood cell count of 17750/μL and a CRP level of 17.15 mg/dL. At the time, she had no recollection of ingesting a fish bone. Subsequently, she experienced hemoptysis once or twice a year and was treated with antibiotics under the diagnosis of pneumonia.

Five years after the initial episode, she was hospitalized due to hemoptysis. Repeat CT revealed a suspected intrapulmonary foreign body, and she was referred to our hospital. Her medical history included rheumatoid arthritis, for which she was taking 5 mg of prednisolone daily.

On referral, her vital signs were stable, and the hemoptysis had subsided. Laboratory tests showed a mildly elevated white blood cell count of 13280/μL, normal CRP (0.16 mg/dL), and a hemoglobin level of 11.5 g/dL, indicating mild anemia.

**Fig. 1:**
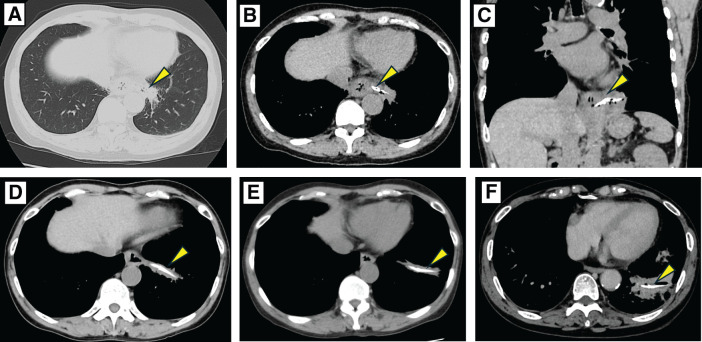
CT images demonstrate peripheral migration of a fish bone from the esophagus into the left lower lobe (LLL). Initial CT at the referring hospital (lung window) shows parahilar consolidation in the LLL (**A**). Corresponding mediastinal window images reveal a linear high-attenuation foreign body measuring approximately 4 cm, extending from the esophagus toward the LLL (**B**, **C**). Follow-up CT at 3 months shows distal migration of the foreign body within the lung parenchyma (**D**), and further progression toward the periphery is noted on the 1-year follow-up CT (**E**). Five years after the initial presentation, CT performed at our institution shows the foreign body lodged in the peripheral LLL, surrounded by a small cavity and adjacent consolidation (**F**). (Arrowheads indicate the foreign body.)

Review of prior CT images revealed a linear, hyperdense structure measuring approximately 4 cm, suspected to be a fish bone, which had penetrated the esophageal wall and migrated into the lung above the diaphragm (**Fig. 1A**–**1C**). There was no evidence of pneumomediastinum, fluid collection, pneumothorax, or pleural effusion. Follow-up CT scans 3 months (**Fig. 1D**) and one year later (**Fig. 1E**) showed gradual migration of the foreign body toward the peripheral lung. CT at our hospital (**Fig. 1F**) revealed a small cavitary lesion and surrounding consolidation at the site of the foreign body.

The object was located in the peripheral lung, and bronchoscopic removal was deemed unfeasible. Therefore, VATS was selected. Although there was some surrounding consolidation, likely due to bleeding, no abscess was apparent. If the foreign body could be identified intraoperatively, we planned to avoid lung resection and perform direct extraction via parenchymal incision. However, if identification was unsuccessful or if inflammation or bleeding was uncontrollable, lung resection would be considered as a second-line strategy.

**Fig. 2:**
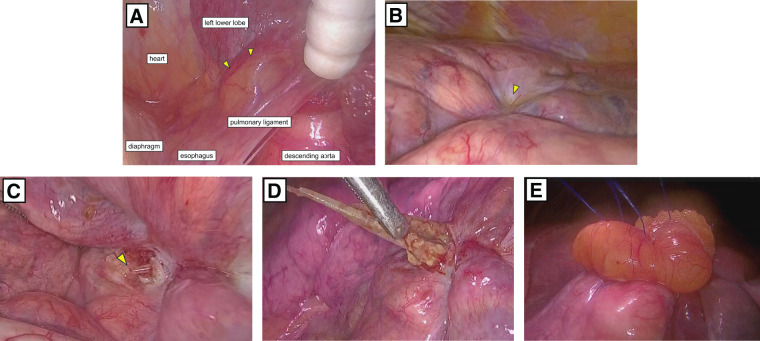
Thoracoscopic observation revealed partial thickening of the pulmonary ligament (arrowheads) (**A**). A localized pleural indentation was observed on the surface of the visceral pleura (arrowhead) (**B**). Upon incision of this area, the tip of the fish bone became exposed (arrowhead) (**C**). The fish bone was carefully extracted using gentle traction (**D**). The lung incision site was closed and reinforced using pericardial fat tissue (**E**).

A lateral thoracotomy approximately 8 cm in length was performed at the 7th intercostal space, and thoracoscopic observation revealed minimal intrathoracic adhesions and partial thickening of the pulmonary ligament (**Fig. 2A**). These findings suggested the possibility that the fish bone had penetrated from the esophagus into the lung through this region. In addition, a localized pleural indentation was observed on the surface of the left lower lobe (**Fig. 2B**), which corresponded to the pleural indentation adjacent to the foreign body seen on CT imaging. On palpation, a firm object was felt just beneath the surface, and a 5-mm incision was made over the area. The fish bone was exposed immediately beneath (**Fig. 2C**) and carefully extracted with forceps without lung resection (**Fig. 2D**). There was almost no adhesion or bleeding during the procedure, even after incising the lung and removing the fish bone. The lung incision site was closed and reinforced using pericardial fat tissue, which was sutured with 4-0 Prolene. Fibrin glue was not used (**Fig. 2E**).

**Fig. 3:**
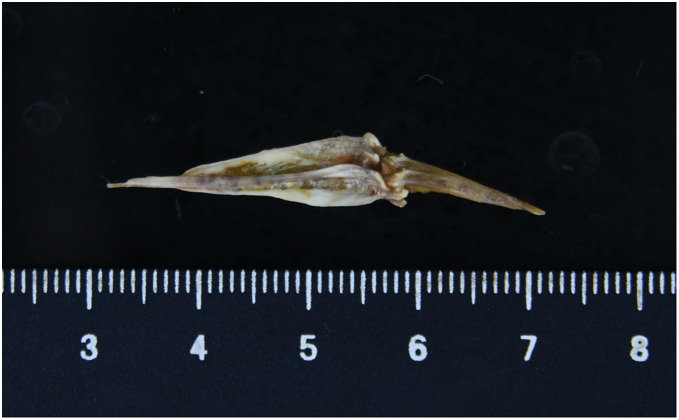
The extracted foreign body was a 4-cm fish bone, which retained its original shape despite having remained in the body for 5 years.

**Fig. 4:**
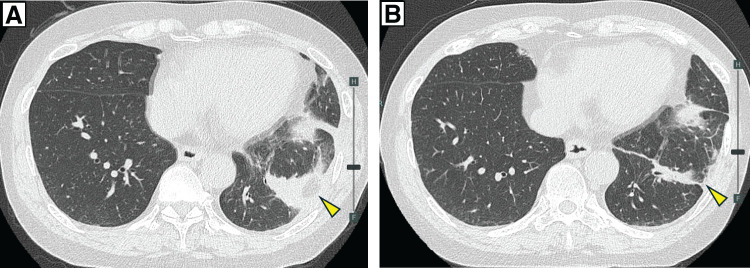
Follow-up CT at 1 month (**A**) and 4 months (**B**) postoperatively shows no residual foreign body, abscess, or granulomatous lesion. The surrounding consolidation had nearly resolved. The sutured pericardial fat tissue (arrowheads) appeared stable at 1 month but had decreased in size by 4 months.

The extracted object was a 4-cm-long fish bone (**Fig. 3**). The postoperative course was uneventful, with no recurrence of hemoptysis. The chest drain was removed on POD 1, and the patient was discharged on POD 9. Follow-up CT at 1 and 4 months postoperatively showed no residual foreign body, abscess, or granulomatous formation, and the surrounding consolidation had nearly resolved (**Fig. 4A**, **4B**).

## DISCUSSION

This is an extremely rare case of a fish bone that penetrated the esophageal wall and migrated along the pulmonary ligament into the peripheral lung. Although foreign body ingestion is relatively common, approximately 80%–90% of cases resolve spontaneously, 10%–20% require endoscopic intervention, and less than 1% necessitate surgical removal.^1,4)^ Among these, sharp objects such as fish bones may perforate the gastrointestinal tract and lead to severe complications.

The esophagus, lacking a serosal layer, is particularly vulnerable to perforation, which can result in mediastinitis or vascular injury if the foreign body enters the mediastinum.^2,5)^ Li et al. reported that among 127 cases of esophageal fish bone penetration, 8 (6.3%) developed mediastinal infection, and another 8 (6.3%) suffered major vascular injury.^3)^ These complications can be life-threatening, highlighting the importance of early recognition and appropriate management.

Migration into the lung or bronchus is extremely rare; in the same report, only 4 cases (3.1%) involved pulmonary or bronchial penetration.^3)^ Reports of fish bones penetrating the esophagus and migrating into the lung are extremely rare. To our knowledge, including the present case, only 5 such cases have been reported (**Table 1**).^6–9)^ In the 4 previously reported cases, the diagnosis and treatment were completed within 30 days after esophageal penetration.

**Table 1:** Reported cases of fish bone penetration of the esophagus with migration into the lung

Author	Year	Country	Age/Sex	Interval from ingestion to diagnosis	Clinical presentation	Imaging findings	Location	Treatment
Fujino et al.^6)^	2012	Japan	55/F	16 days	Fever, chest pain	Fish bone penetrating esophagus into right lower lobe	RLL	VATS removal
Matsuda et al.^8)^	2013	Japan	60/F	3 days	Fever, chest pain	Lung abscess near esophagus	RUL	Lobectomy
Tan et al.^7)^	2015	China	47/M	20 days	Cough, fever	Fish bone penetrating upper esophagus into lung	RUL	Lung Partial resection
Tuan et al.^9)^	2024	Vietnam	63/M	1 month	Hemoptysis, chest pain	Fish bone penetrating esophagus into right pulmonary artery wall	RUL	Open + endoscopic removal
Our case	2025	Japan	80/F	5 years	Hemoptysis	Fish bone migrating into the lung	LLL	VATS removal

F, female; LLL, left lower lobe; M, male; RLL, right lower lobe; RUL, right upper lobe; VATS, video-assisted thoracoscopic surgery

In our case, the fish bone appeared to have penetrated the esophagus and migrated along the pulmonary ligament into the peripheral lung—a pathway rarely documented in the literature. A similar case was reported by Fujino et al., in which a fish bone entered the right pulmonary ligament and was removed thoracoscopically.^6)^ However, in their case, the fish bone remained within the pulmonary ligament and could be extracted by incising the ligament itself. To the best of our knowledge, our case represents the only reported instance in which a fish bone traversed the pulmonary ligament and migrated into the peripheral lung parenchyma, making it an exceptionally rare presentation.

Although bacterial pneumonia was suspected at the initial presentation, retrospective evaluation revealed that the inflammatory findings were more consistent with a localized reaction to the retained fish bone. Long-term retention of foreign bodies in the lung may lead to chronic inflammation, fistula formation, or granulomatous lesions.^10)^ In this case, cavitary changes and surrounding consolidation were noted, but no abscess or mass formation was observed. Given the peripheral location of the foreign body, bronchoscopy was considered inappropriate, and surgical extraction via thoracoscopy was planned.

The method of extraction depends on the object’s size, shape, location, and relationship with the surrounding structures. In cases where endoscopic removal is not feasible, surgical intervention is necessary. Previous reports have described lobectomy or segmentectomy for similar cases involving long-term retained fish bones, particularly when accompanied by severe infection or tissue damage.^8,11,12)^

In contrast, our case presented with only localized consolidation, likely due to mild bleeding or inflammation. Hemoptysis was the main symptom, and we judged that simple removal of the foreign body would suffice. Even if a pulmonary abscess had been present, we considered it manageable with antibiotics. In the event of recurrent infection or hemoptysis, lung resection could be considered electively.

The postoperative course was favorable, and CT at 4 months showed near-complete resolution of inflammation without recurrence of symptoms. We plan to continue regular imaging follow-up to monitor for delayed sequelae, but at present, complete resolution has been achieved with local removal alone.

This case represents a highly unusual and clinically significant scenario in which a fish bone penetrated the esophagus, remained in the lung for an extended period, and was successfully removed via a minimally invasive approach without lung resection. The ability to visualize the migration trajectory through serial imaging and to avoid extensive surgery underscores the diagnostic and therapeutic value of careful follow-up. In cases of unexplained pulmonary lesions or persistent respiratory symptoms, the possibility of a retained foreign body should always be considered. Furthermore, when surgical removal is required, localized extraction without lung resection may offer an effective and less invasive treatment option.

## CONCLUSIONS

We report an extremely rare case of a fish bone that penetrated the esophageal wall and migrated into the peripheral lung parenchyma, remaining undiagnosed for 5 years. Despite the long duration and the presence of local inflammation, the foreign body was successfully removed via VATS without the need for lung resection. This case highlights the importance of considering retained foreign bodies in the differential diagnosis of unexplained pulmonary symptoms and imaging findings. Furthermore, it demonstrates that even long-standing foreign bodies may be managed effectively with a minimally invasive, lung-preserving approach when preoperative evaluation is carefully conducted.

## References

[1] Birk M, Bauerfeind P, Deprez PH, et al. Removal of foreign bodies in the upper gastrointestinal tract in adults: European Society of Gastrointestinal Endoscopy (ESGE) Clinical Guideline. Endoscopy 2016; 48: 489–96. 26862844 10.1055/s-0042-100456

[2] Akaishi T, Ishizawa K, Fukutomi T, et al. Penetration of a swallowed fish bone into pulmonary vein: diagnosis and management. Heliyon 2020; 6: e05611. 33294720 10.1016/j.heliyon.2020.e05611 PMC7701346

[3] Li D, Zeng WT, Chen JC. Fish bone migration: complications, diagnostic challenges, and treatment strategies. World J Emerg Surg 2025; 20: 35. 40269939 10.1186/s13017-025-00611-9 PMC12016485

[4] Eisen GM, Baron TH, Dominitz JA, et al. Guideline for the management of ingested foreign bodies. Gastrointest Endosc 2002; 55: 802–6. 12024131 10.1016/s0016-5107(02)70407-0

[5] Zhong S, Wu Z, Wang Z. Successful treatment of fishbone-induced esophageal perforation and mediastinal abscess: a case report and literature review. Am J Case Rep 2023; 24: e942056. 38105546 10.12659/AJCR.942056 PMC10740317

[6] Fujino K, Mori T, Yoshimoto K, et al. Esophageal fish bone migrating to the lung: report of a case (in Japanese). Kyobu Geka 2012; 65: 922–5. 22940666

[7] Tan S, Tan S, Peng M, et al. Management of an ingested fish bone in the lung using video-assist thoracic surgery: a case report. Medicine (Baltimore) 2015; 94: e943. 26039134 10.1097/MD.0000000000000943 PMC4616352

[8] Matsuda E, Okabe K, Takahagi A, et al. Pulmonary abscess caused by fish bone (in Japanese). Kyobu Geka 2013; 66: 219–22. 23445648

[9] Tuan HX, Hung ND, Quang NN, et al. Pulmonary artery penetration due to fish bone ingestion: a rare case report. Radiol Case Rep 2024; 19: 1900–6. 38425774 10.1016/j.radcr.2024.02.003 PMC10904187

[10] Ohshimo S, Guzman J, Costabel U, et al. Differential diagnosis of granulomatous lung disease: clues and pitfalls. Eur Respir Rev 2017; 26: 170012. 28794143 10.1183/16000617.0012-2017 PMC9488688

[11] Laguna S, Lopez I, Zabaleta J, et al. Actinomycosis associated with foreign body simulating lung cancer. Arch Bronconeumol 2017; 53: 284–285. 27816277 10.1016/j.arbres.2016.09.006

[12] Pan S, Chai Y, Shen G. Recurrent pneumonia caused by a migrated esophageal foreign body. Thorac Cardiovasc Surg 2013; 61: 513–515. 23212158 10.1055/s-0032-1331039

